# Three-Dimensional Innovations in Personalized Surgery

**DOI:** 10.3390/jpm13010113

**Published:** 2023-01-04

**Authors:** Joep Kraeima, Sebastiaan de Visscher, Max Witjes

**Affiliations:** 1 Department of Oral and Maxillofacial Surgery, University of Groningen, University Medical Center Groningen, 9713 GZ Groningen, The Netherlands; 2 3D Lab, University Medical Center Groningen, University of Groningen, 9713 GZ Groningen, The Netherlands

Due to the introduction of three-dimensional (3D) technology in surgery, it has become possible to preoperatively plan complex bone resections and reconstructions, (corrections and adjustments related to bones), from head to toe. Three-dimensional technology has proven to be a valuable tool for the surgeon, especially when executing complex surgery in the operating room, as crucial decision making with regard to resection margins, planning of osteotomies, screw, and dental implant location is predetermined by virtual planning [1].

Dedicated 3D virtual surgical planning (VSP) software gives a detailed 3D virtual model of the patient based on CT and MRI scans or other imaging modalities, in order to measure, evaluate, simulate or correct parameters that are relevant to the treatment.

This 3D VSP workflow has evolved from a supporting visualization and virtual measurement and evaluation tool to an integrated method that allows for complete pre-operative surgical decision making and designing patient specific implants (designed for surgical procedures) [2].

The use of 3D virtual planning, 3D printing of surgical aids (and parts), as well as navigational technology, is associated with the adage ‘plan your operation and operate your plan’ [1,3]. The increasing availability and useability of the 3D software and translation instruments, such as 3D-printed guides, has led to the widespread use of some form of 3D technology in healthcare. This has led to improvements in terms of accuracy, predictability and safety for both the surgeon and the patient.

*The Next* *Step*

The workflow of 3D VSP and subsequent design of patient-specific implants (PSI) have evolved in recent years as a result of automation and developments in printing.

Automation of the 3D VSP steps can be achieved (as reported in the literature) by means of new segmentation software tools, artificial intelligence applications and other application-specific optimization methods. This leads to faster and less user-dependent preparation of a 3D VSP [4].

Recent developments in the field of 3D printing allow us to develop more complex designs of patient-specific implants, use different materials (for the implants) and optimize the implants’ surface. Application of biomechanical models and finite element methods can predict the behavior of, e.g., osteosynthesis plates or implants in a patient, and therefore can be used to improve the design of osteosynthesis materials and implants. In search of further optimization of the design of 3D VSP-based osteosynthesis materials and implants, the output of a FE model should be applied in the design process by means of a topology optimization (TO) process.

This Special Issue, entitled ‘3D innovations in personalized surgery’, presents a series of highly innovative studies and reviews on bone-related applications of the latest 3D technology. These applications include optimization of the 3D VSP, developments in patient-specific biomechanical modeling, inclusion of motion (4D), implant optimizations, surgical navigation and post-operative evaluation of accuracy.

Three-dimensional technology has become the standard-of-care and is expected to bring many more advantages for both the surgeon and the patient in the near future.
